# Computed tomography measurement of glenoid vault version as an alternative measuring method for glenoid version

**DOI:** 10.1186/1749-799X-9-17

**Published:** 2014-03-11

**Authors:** Noboru Matsumura, Kiyohisa Ogawa, Hiroyasu Ikegami, Philippe Collin, Gilles Walch, Yoshiaki Toyama

**Affiliations:** 1 Department of Orthopedic Surgery, School of Medicine, Keio University, 35 Shinanomachi, Shinjuku-ku, Tokyo 160-8582, Japan; 2 Department of Orthopedic Surgery, Eiju General Hospital, 2-23-16 Higashi-Ueno, Taito-ku, Tokyo 110-8645, Japan; 3 Department of Orthopedic Surgery, Toho University Ohashi Medical Center, 2-17-6 Ohashi, Meguro-ku, Tokyo 153-8515, Japan; 4 Centre Hospitalier Privé Saint-Grégoire, 6 boulevard de la Boutière, Saint-Grégoire, Rennes 35740, France; 5 Départment de Chirurgie Orthopédique, Centre Orthopédique Santy et Hôspital Privé Jean Mermoz, 24 Avenue Paul Santy, Lyon 69008, France

**Keywords:** Glenoid vault, Glenoid version, Glenoid retroversion, Shoulder arthroplasty, Glenoid component, Glenoid replacement, Glenoid morphology

## Abstract

**Background:**

The conventional measuring method for glenoid version is greatly influenced by the scapular body shape that varies widely between patients. We postulated that the glenoid vault version could be more useful than the conventional glenoid version in clinical cases.

**Objectives:**

The purposes of this study were to compare the values of glenoid version measured with the conventional method to those with the vault method and to investigate the feasibility of the glenoid vault version.

**Methods:**

Computed tomography scans of 150 normal shoulders and 150 arthritic shoulders were analyzed. Three-dimensionally corrected slices were reconstructed from the Digital Imaging and Communications in Medicine (DICOM) data, and glenoid version was measured with both the conventional and vault methods. After determining intra- and interrater reliabilities, differences in glenoid version values between the conventional and vault methods were assessed. In the normal shoulder group, side-to-side differences of glenoid version values were also evaluated in both methods.

**Results:**

Both measuring methods demonstrated high intra- and interrater reliabilities. The normal glenoid had 1.1° ± 3.2° retroversion with the conventional method and 8.9° ± 2.7° retroversion with the vault method. The average glenoid retroversion of arthritic shoulders was 10.8° ± 9.3° measured with the conventional method and 18.2° ± 9.1° with the vault method. The vault method showed significantly larger glenoid retroversion than the conventional method in both normal and arthritic shoulder groups. Both conventional glenoid retroversion and glenoid vault retroversion were significantly larger on dominant sides than on nondominant sides in the normal shoulders.

**Conclusions:**

The glenoid vault version could be used as an alternative measuring method for glenoid version with high reliability. In clinical use, the glenoid vault version appears to be more useful than the conventional glenoid version to assess the severity of arthritis and difficulty of glenoid replacement. The glenoid vault is not symmetric, but usually retroverted in both normal and arthritic shoulders.

## Introduction

Proper recognition of the individual glenoid version is important for anatomical reconstruction in shoulder arthroplasty [1-3]. Computed tomography (CT) evaluation is the most popular method for the measurement of glenoid version in clinical cases [4-8]. The conventional measuring method for glenoid version described by Friedman et al. has been widely utilized [4], and normal shoulders are expected to have neutral version in accordance with a small number of past studies [4,5,9]. However, the conventional method is sensitive to the scapular body shape that varies widely between patients. The conventional glenoid version should be considered as a ‘scapular version’ rather than a ‘glenoid version,’ and we propose to use the glenoid vault method as an alternative measuring method for glenoid version in order to exclude the influence of the scapular body shape and to attain more accurate values.

We hypothesized that the conventional measuring method and the glenoid vault method would give different values of glenoid version and that the glenoid vault would have larger retroversion than had previously been thought. The purposes of this study were to compare the values of glenoid version measured with the two methods and to assess the feasibility of the glenoid vault version.

This study was approved by the Institutional Review Board of Centre Orthopédique Santy et Hôspital Privé Jean Mermoz (reference study number 45).

### Patients and methods

Both normal and pathological shoulders were examined in this study. The normal shoulder group prospectively included axial CT scans of 150 shoulders from 75 healthy volunteers ranging from 20 to 30 years of age (mean age, 26.5 ± 2.6 years; 38 males and 37 females). All volunteers gave informed consent to participate in this study, and candidates with any past illnesses or injuries in the shoulder girdles were excluded. All of them were engaged in light work, and no one performed heavy labor or competitive sports activity at the time of investigation. The dominant shoulders were right in 69 and left in 6. Bilateral CT scans with 3-mm-thick contiguous glenoid slices were taken with the elbow extended, the forearm supinated, and the palm fixed under the buttock to prevent arm motion (GE Healthcare HiSpeed NX/i Pro, Amersham, England, or Toshiba Aquilion^TM^ TSX-101A, Tokyo, Japan). The pathological shoulder group consisted of a retrospective review of 150 shoulders from 150 consecutive patients with primary glenohumeral arthritis (mean age, 68.4 ± 9.0 years; range, 41–89 years; 55 males and 95 females) who had unilateral CT arthrograms (Philips MX 8000, IDT 16, Amsterdam, Netherlands). The shoulders with previous surgeries involving the scapular region were excluded. The right shoulder was involved in 85 patients, and the left was involved in 65 patients. Thirty-four shoulders were classified using the Walch classification scheme [10] as type A1, 30 as type A2, 41 as type B1, 39 as type B2, and 6 as type C. All of the shoulders were positioned with neutral shoulder position during the examination. CT scans were taken with 0.4-mm-thick contiguous slices of the glenoid.

**Figure 1:**
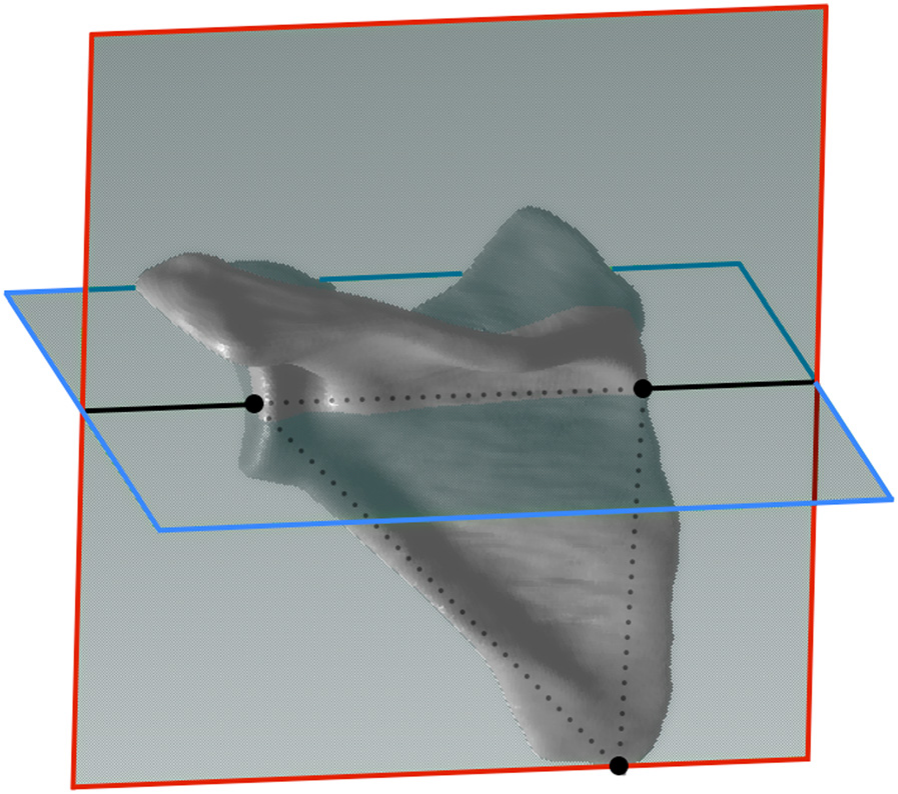
**Reconstruction of three-dimensionally corrected slice.** The inferior tip of the scapular body, the center of the glenoid surface, and the root of the scapular spine (the *triangle* made by the *black dotted lines*) are selected to determine the scapular plane (the plane surrounded by *red lines*). The plane including the scapular axis, which is defined as the line connecting the root of the scapular spine and the center of the glenoid surface (the *black solid lines*), and perpendicular to the scapular plane is reconstructed as the three-dimensionally corrected slice (the plane surrounded by *blue lines*).

**Figure 2:**
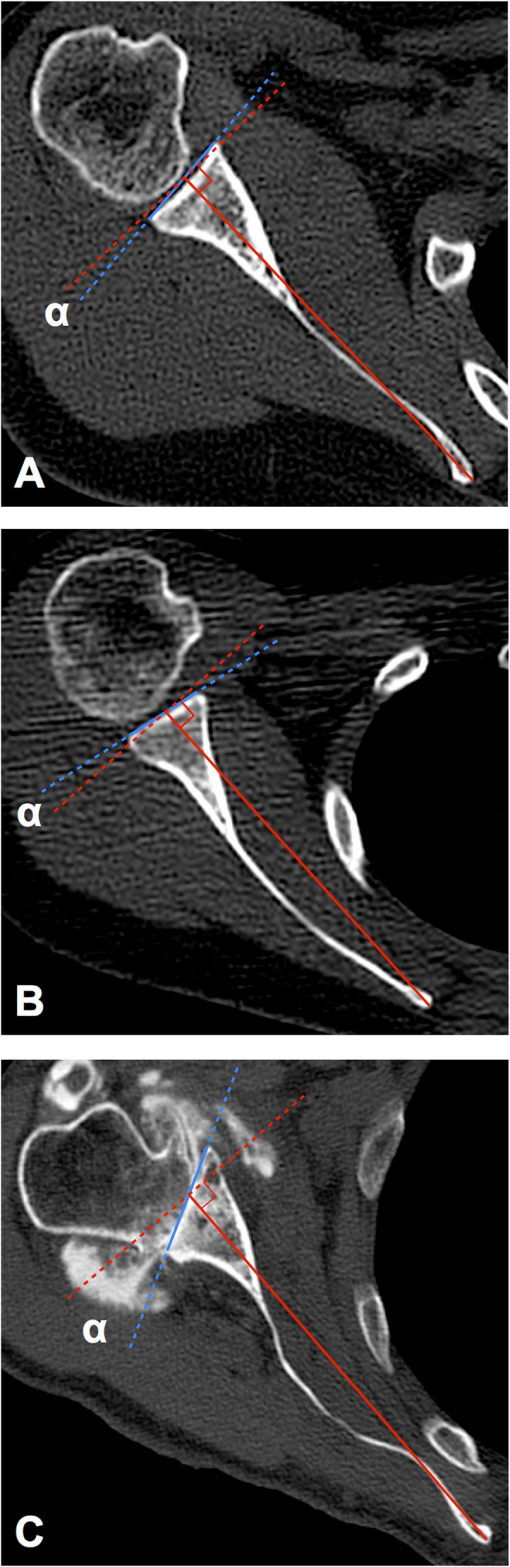
**The conventional method for glenoid version measurement.** The figures describe the conventional measuring method for glenoid version described by Friedman et al. The glenoid line is defined as the line connecting the anterior and the posterior rim of the glenoid (*blue line*). The scapular axis is defined as the line connecting the root of the scapular spine and the center of the glenoid line (*red solid line*). Glenoid version (*α*) is calculated as the angle between the glenoid line and the line perpendicular to the scapular axis (*red dashed line*). **(A)** The conventional method for measuring a normal shoulder. The scapular body shape appears flat. The glenoid shows 6.2° retroversion. **(B)** The conventional method for another normal shoulder. The scapular body shape is round. The glenoid shows 8.9° anteversion. The conventional method is sensitive to the scapular body shape that varies widely between patients. **(C)** The conventional method for measuring an arthritic shoulder. The scapular body is waving. CT arthrograms are used to measure glenoid version, and the glenoid has 28.3° retroversion. The intermediate line is selected as the glenoid line in the Walch classification B2 glenoid.

**Figure 3:**
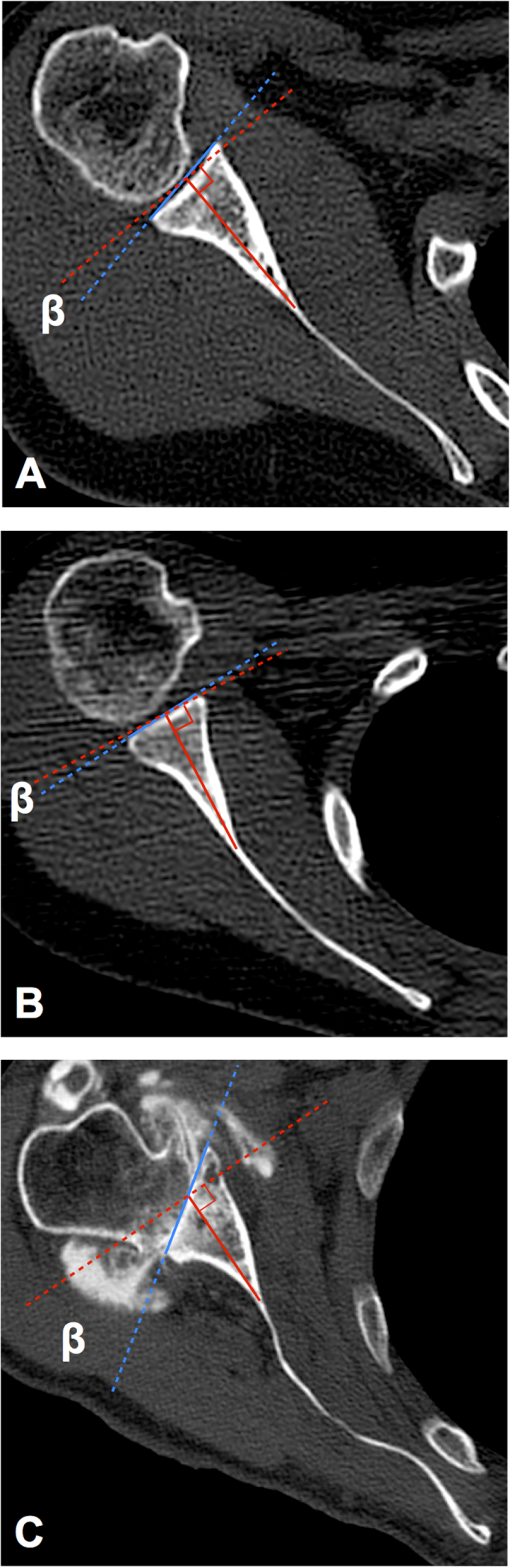
**The vault method as an alternative glenoid version measurement.** The same cases as Figure 2 are measured with the vault method. The glenoid line is the same as that of the conventional method (*blue line*). The glenoid vault axis is defined as the line connecting the tip of the scapular vault and the center of the glenoid line (*red solid line*). Glenoid version measured with the vault method (*β*) is calculated as the angle between the glenoid line and the line perpendicular to the glenoid vault axis (*red dashed line*). **(A)** The vault method for measuring a normal shoulder. Glenoid vault retroversion is 9.7°. The difference between conventional glenoid version and glenoid vault version is only 3.5°. **(B)** The glenoid has 5.0° retroversion when measured with the vault method. The difference between conventional glenoid version and glenoid vault version amounts to 13.9°. **(C)** The vault method for measuring an arthritic shoulder. The glenoid vault has 34.8° retroversion. The difference between conventional glenoid version and glenoid vault version is 6.5°.

The obtained Digital Imaging and Communication in Medicine (DICOM) data were analyzed using the Osirix MD 1.4.1 software (Pixmeo, Geneva, Switzerland). In order to exclude the effect of scapular inclination, three-dimensionally corrected slices were reconstructed on the software [1,11]. We determined the scapular plane as the plane including the inferior tip of the scapular body, the center of the glenoid surface, and the medial pole of the scapula [12]. The three-dimensionally corrected slice was reconstructed as the plane including the center of the glenoid surface and the medial pole of the scapula, and perpendicular to the scapular plane [13] (Figure 1). We examined glenoid version with the conventional measuring method described by Friedman et al. [4] (conventional method) and the glenoid vault method (vault method). Both methods defined the glenoid line as the line connecting the anterior rim with the posterior rim of the glenoid. The intermediate line was selected as the glenoid line [8] in the Walch classification [10] type B2 glenoid of the arthritic shoulders. The scapular axis was defined as the line connecting the tip of the medial border of the scapula and the center of the glenoid line in the conventional method. Glenoid version measured with the conventional method was calculated as the angle between the glenoid line and the line perpendicular to the scapular axis (Figure 2A,B,C). In the vault method, we defined the glenoid vault axis as the line connecting the tip of the scapular vault and the center of the glenoid line. Glenoid version with the vault method was calculated as the angle between the glenoid line and the line perpendicular to the glenoid vault axis (Figure 3A,B,C). Three evaluators independently reviewed all measurements twice with a minimum of a 1-month interval between measurements. Each measurement started from slice reconstruction.

Statistical analyses were performed using IBM SPSS Statistics 20.0.0 software (IBM, Armonk, NY, USA). Intra- and interrater reliabilities were evaluated with intraclass correlation coefficients (ICCs) first. Glenoid version measurement reliability was examined in the normal and arthritic shoulder groups for both the conventional and vault methods. Intrarater reliability for each of three observers was calculated by repeated measurements with a 1-month interval (ICC model 1.1). Interrater reliability was calculated by blinded measurements of three observers (ICC model 2.1). After reliability was assessed, the glenoid version values were averaged across the three observers and their two measurements. The glenoid version values measured with the two methods were compared with the Wilcoxon signed-rank tests, and their distributions were compared using *F*-tests in both normal and arthritic shoulders. In the normal shoulders, differences of glenoid version values between the dominant and nondominant shoulders were compared using Wilcoxon signed-rank tests in both the conventional method and the vault method. The significance level was set at 0.05 for all analyses.

## Results

Intrarater reliability of the three observers in conventional glenoid version measurement was 0.934 (95% confidence interval (CI), 0.910–0.952), 0.922 (95% CI, 0.894–0.943), and 0.904 (95% CI, 0.881–0.923) for normal shoulders and 0.976 (95% CI, 0.958–0.986), 0.959 (95% CI, 0.929–0.976), and 0.919 (95% CI, 0.861–0.953) for arthritic shoulders, respectively. Intrarater reliability of glenoid vault version was 0.901 (95% CI, 0.866–0.927), 0.914 (95% CI, 0.854–0.950), and 0.895 (95% CI, 0.859–0.923) for normal shoulders and 0.903 (95% CI, 0.835–0.944), 0.923 (95% CI, 0.869–0.956), and 0.930 (95% CI, 0.878–0.960) for arthritic shoulders, respectively. Interrater reliability of the conventional method was scored as 0.939 (95% CI, 0.920–0.954) for normal shoulder measurement and as 0.953 (95% CI, 0.936–0.966) for arthritic shoulder measurement. Interrater reliability of the vault method was 0.908 (95% CI, 0.867–0.937) for normal shoulder measurement and 0.905 (95% CI, 0.871–0.930) for arthritic shoulder measurement. Intra- and interrater reliabilities exceeded 0.85 for glenoid version measurements with both measuring methods in all analyses.

**Figure 4:**
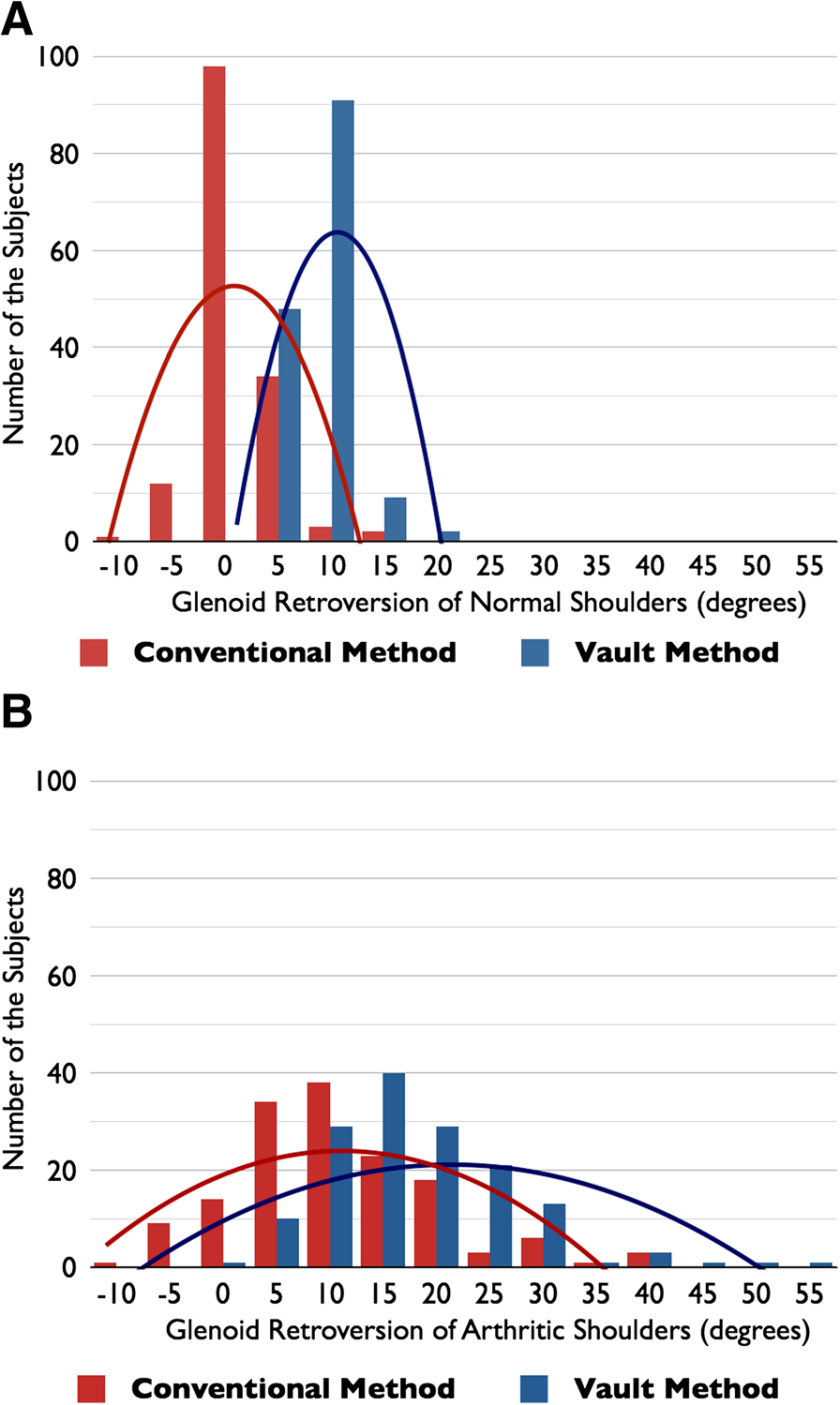
**The histogram of glenoid version measured with the two different methods. (A)** The histogram of glenoid version in the normal shoulders. The values measured with the conventional method are concentrated between -5° and 5°, and those with the vault method are between 5° and 15°. The distributions of the values of version are significantly larger with the conventional method than with the vault method (*p* = 0.030). **(B)** The histogram of glenoid version measured in the arthritic shoulders. The values are widely distributed from -8.8° to 42.3° with the conventional method and from 1.4° to 56.8° with the vault method. The distributions of the values of version are not different between the conventional method and the vault method in the arthritic shoulder group (*p* = 0.737).

**Figure 5:**
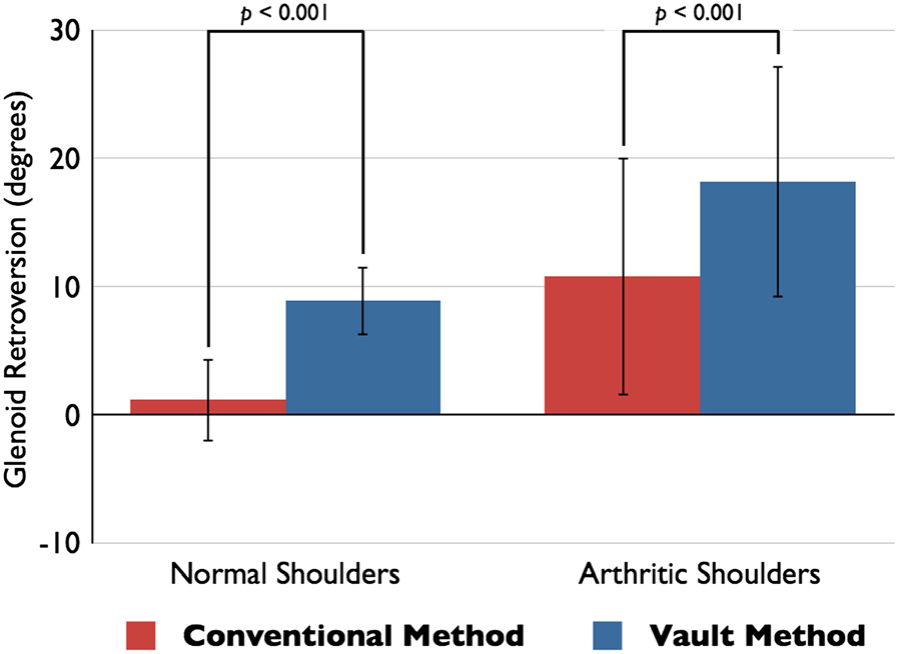
**The average values of glenoid retroversion measured with the two different methods.** The average glenoid retroversion measured with the conventional method and the vault method. The vault method shows significantly larger retroversion than the conventional method in both normal and arthritic shoulders (*p* < 0.001 for both).

The values of glenoid version measured with the conventional method were concentrated between -5° and 5° (range, -8.9° to 16.7°), and those with the vault method were between 5° and 15° (range, 3.2° to 19.7°) (Figure 4A). The distribution of the values of version was significantly larger in the conventional method than in the vault method (*p* = 0.030). A positive number means that there is retroversion, and a negative number indicates that there is anteversion of the glenoid. The average glenoid retroversion was 1.1° ± 3.2° when calculated with the conventional method and 8.9° ± 2.7° when calculated with the vault method for normal shoulders. The retroversion value measured with the vault method was significantly larger than the value measured with the conventional method (*p* < 0.001) (Figure 5). In the normal shoulder group, the dominant shoulder showed larger retroversion of the glenoid than the nondominant side both in the conventional method (1.4° ± 3.4° on the dominant shoulder and 0.8° ± 3.1° on the nondominant shoulder, *p* = 0.013) and in the vault method (9.6° ± 2.7° on the dominant shoulder and 8.2° ± 2.6° on the nondominant shoulder, *p* < 0.001).

In the arthritic shoulder group, the values of glenoid retroversion were widely distributed from -8.8° to 42.3° with the conventional method and from 1.4° to 56.8° with the vault method (Figure 4B). No difference was found in their distributions of arthritic shoulders (*p* = 0.737). The mean glenoid retroversion values of the arthritic shoulder group were larger than the values of the normal shoulder group. The arthritic shoulder glenoid demonstrated a mean of 10.8° ± 9.3° retroversion with the conventional method and a mean of 18.2° ± 9.1° retroversion with the vault method. The vault method showed significantly larger retroversion than the conventional method in the arthritic shoulders (*p* < 0.001) (Figure 5).

## Discussion

Scapular morphology appears to be modular [14]. The scapular body is congruent with the thorax, and together they form the scapulothoracic joint. The glenoid vault is congruent with the humeral head, and together they form the glenohumeral joint. During shoulder arthroplasty, it is important to recognize the respective glenoid version for proper placement of the glenoid component within the vault [1,15,16]. Severe glenoid version is one of the risk factors for postoperative loosening [2,3], and surgical strategy will change with glenoid version [17,18]. However, the conventional method for measuring glenoid version is greatly influenced by the scapular body shape, which widely varies from straight to round and wavy [19]. In the normal shoulder group of this study, the vault method showed a lower standard deviation and different distribution of the values of version compared to the conventional method, and this may be the result of elimination of the variable scapular body effects. Although the arthritic glenoid also showed a lower standard deviation of glenoid vault version than the conventional glenoid version, their distributions were not different. The wide variations in version were supposed to lead the result. As the rotator cuff muscles originate from the medial border of the scapula, conventional glenoid version might represent the force balance of the rotator cuff. Nevertheless, the glenoid vault version, which eliminates the scapular body effect, is likely to be more important for clinical use than the conventional glenoid version. The vault method showed significantly larger retroversion than the conventional method in both normal shoulders and arthritis shoulders. The conventional glenoid version could possibly underestimate the severity of arthritis.

We can eliminate the scapular body angulation in measuring glenoid version with the glenoid vault method. We can also check the respective glenoid vault shape by inspecting the anterior wall of the vault during arthroplasty, but it will be impossible to assess the conventional version that utilizes the medial border of the scapula. Finally, the glenoid vault version can be evaluated even if the CT scan does not include the entire scapula, which unfortunately happens quite frequently. The vault method showed high intra- and interrater reliabilities in both normal and arthritic shoulders as well as the conventional method. Thus, glenoid vault version can be used as an alternative measuring method for glenoid version. Poon and Ting also focused on the glenoid vault in version measurement, and they reported their original measuring method using an isosceles triangle pictured within the medial end of the glenoid endosteal vault [19]. However, it is important for proper placement of the glenoid component to understand the location of the glenoid vault with respect to the glenoid center. During glenoid replacement, we face the glenoid surface and not the glenoid endosteal vault. In clinical use, we believe that our glenoid vault version could be more useful than their measuring method to assess the severity of arthritis and difficulty of shoulder arthroplasty.

As the scapula is a three-dimensional structure, two-dimensional measurement of glenoid version might be influenced by the scapular position and the gantry angle of the CT scans [11,13]. Furthermore, the glenoid is known to twist anteriorly to posteriorly [20,21], and slice selection can change the values of version. For these reasons, we reconstructed three-dimensionally corrected slices to clarify the accurate values of conventional glenoid version and glenoid vault version. Our study is the first computed tomographic analysis of glenoid vault version with three-dimensionally reconstructed slices. In the normal shoulders, the glenoid has almost neutral version when measured with the conventional method and a mean of 9° retroversion with the vault method. The normal glenoid vault is not symmetric, but usually retroverted. The present study also revealed side-to-side differences in glenoid version and glenoid vault version. In the normal shoulders, the dominant side had significantly larger glenoid retroversion than the nondominant side with both the conventional method and the vault method. Crockett et al. reported that professional baseball pitchers had significantly larger glenoid retroversion in dominant shoulders than in nondominant shoulders [22]. The present result was consistent with the past study, and the glenoid is thought to be retroverted in highly demanding situations. The differences between sides appear to occur in the glenoid vault and not in the scapular body.

The 3-mm-interval thickness of CT scans in the normal shoulder group can be a limitation of the present study. The thickness was selected to minimize exposure to radiation because the normal shoulder group dealt with healthy volunteers. More detailed scans might bring more precise results, but our version measurement showed good-to-excellent intra- and interrater reliabilities even with this thickness. Thus, the results appeared to be reliable.

## Conclusions

The present study revealed that the glenoid vault method could be clinically utilized as an alternative measuring method for glenoid version in both normal and pathological shoulders. Our results indicated that the glenoid vault is not neutral, but usually retroverted without the influence of the scapular body shape. Dominant shoulders had significantly larger glenoid retroversion than nondominant shoulders, and the side-to-side difference appears to occur in the glenoid vault and not in the scapular body.

## Competing interests

The authors declare that they have no competing interests.

## Authors' contributions

NM conceived of the study, analyzed the collected data, and drafted the manuscript. KO and GW recruited the subjects and assembled the database. HI and PC analyzed the collected data. YT supervised the writing of the paper and gave final approval. All authors read and approved the final manuscript.
